# Successful surgical procedure based on careful preoperative imaging for chronic idiopathic colonic pseudo-obstruction: a case report

**DOI:** 10.1186/s40792-020-01048-9

**Published:** 2020-10-28

**Authors:** Kosuke Yoshimura, Hiroki Ohge, Norimitsu Shimada, Shinnosuke Uegami, Yusuke Watadani, Ikki Nakashima, Toshinori Hirano, Hiroki Kitagawa, Yuki Kaiki, Shinya Takahashi

**Affiliations:** 1 Department of Surgery, Graduate School of Biomedical & Health Sciences, Hiroshima University, 1-2-3 Kasumi, Minami-Ku, Hiroshima, Hiroshima 734-8551 Japan; 2 Department of Surgery, National Hospital Organization Kure Medical Center and Chugoku Cancer Center, 3-1 Aoyama, Kure, Hiroshima 737-0023 Japan

**Keywords:** Chronic idiopathic colonic pseudo-obstruction, Laparoscopic surgery, Total colectomy, Ileo-rectal anastomosis

## Abstract

**Background:**

Chronic idiopathic colonic pseudo-obstruction (CICP) is a rare disease, defined as a condition of the chronically damaged colon, without obstruction or stenosis, and a pathological abnormality in the myenteric plexus. To date, there is no effective medication for CICP, and existing medication is not useful, making surgery the only effective treatment. Laparoscopic surgery is useful for reducing surgical trauma and postoperative adhesion. Herein, we report a patient with recurrent laxative-uncontrolled bowel obstruction, who underwent successful treatment with laparoscopic total colectomy based on preoperative detailed evaluation of bowel function.

**Case presentation:**

A 77-year-old female patient without any past abdominal or psychological medical history was referred to our hospital because of chronic constipation and abdominal pain. Contrast-enhanced computed tomography, barium enema, cine magnetic resonance imaging, and defecography indicated an enlarged colon from the cecum to the transverse colon (proximal to the splenic flexure) without apparent mechanical obstruction, and a collapsed colon from the descending colon to the rectum, with reduced peristalsis. Bowel movements of the rectum and anorectal function were normal. Based on these findings, we diagnosed CICP and performed laparoscopic total colectomy and ileo-rectal anastomosis in this case. Postoperative recovery was good, without the need for postoperative laxatives. Pathologically, no degeneration of the muscle layers or Auerbach’s plexus was found in the resected specimen.

**Conclusion:**

Surgery is the only effective treatment for patients with CICP. Careful imaging before surgery is important for detecting the extent of excision required. This will reduce the need for additional surgery due to symptom relapse in the remnant colon. However, continued observation of the patient is required.

## Background

Chronic idiopathic colonic pseudo-obstruction (CICP) occurs in a chronically damaged colon without obstruction or stenosis, and defective peristalsis is limited to the colon.

For the definitive diagnosis of CICP, we need to exclude diseases such as Hirschsprung’s disease or acquired isolated hypoganglionosis, which are associated with abnormalities in the myenteric plexus, and occur secondary to systemic disease or medication, respectively. To date, there is no effective medication for CICP, and surgery is the only treatment. It is important to detect the range of bowel excision precisely based on several preoperative examinations, for improving symptoms, avoiding recurrence, and also maintaining bowel function. Herein, we report a patient with recurrent laxative-uncontrolled bowel obstruction, who underwent successful treatment with laparoscopic total colectomy based on preoperative detailed evaluation of bowel function.

## Case presentation

**Fig. 1:**
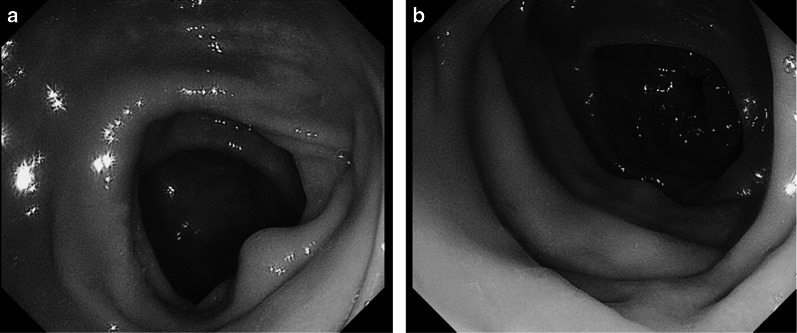
Total colonoscopy. Although mucosal edema is observed in several portions, no mechanical obstruction or stenosis was found from the cecum (**a**) to the rectum (**b**)

A 77-year-old female patient was referred to our hospital because of chronic constipation and abdominal pain. Her abdominal and psychological medical history was normal. She had experienced severe constipation for 2 years and required hospitalization several times for bowel obstruction without apparent mechanical obstruction. She was prescribed several laxatives including magnesium, mosapride citrate hydrate, pantethine, lubiprostone, daikenchuto, and sodium picosulfate hydrate. We performed colonoscopy, and the stored gas and stool in the enlarged colon were evacuated to prevent ileus. Similar to findings from previous investigations, no mechanical obstruction or stenosis was found from the cecum to the rectum, although mucosal edema was observed in several portions (Fig. 1).

**Fig. 2:**
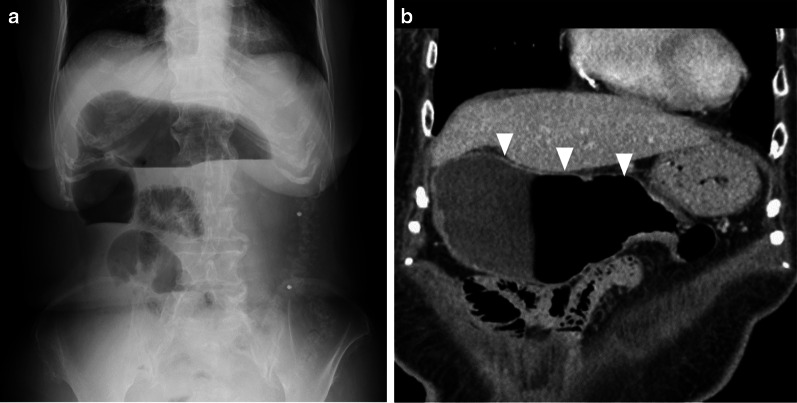
Abdominal X-ray and contrast-enhanced computed tomography (CT). **a** X-ray shows massive colon gas niveau formation in the epigastric region. **b** Dilation of the cecum, ascending colon, and transverse colon proximal to the splenic flexure seen on the contrast-enhanced CT scan. No mechanical obstruction is present (arrowhead)

**Fig. 3:**
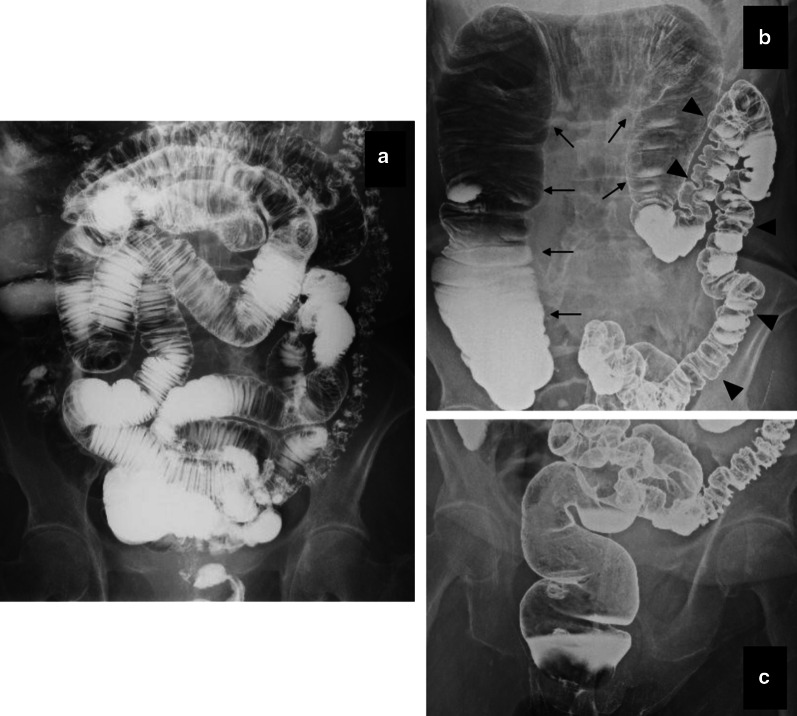
Enteroclysis and barium enema. **a** No stricture, enlargement, or functional decline seen in the small intestine. **b**, **c** The rectum is not dilated; the dilated right-side colon (arrow) and the collapsed left-side colon (arrowhead) are seen with barium enema

Abdominal X-ray showed a massive colonic gas niveau (Fig. 2a). Contrast-enhanced computed tomography (CT) showed an enlarged but well-enhanced colon from the cecum to the transverse colon proximal to the splenic flexure, without mechanical obstruction (Fig. 2b). Enteroclysis revealed that peristalsis of small intestine was intact, and no strictures or enlargements were observed (Fig. 3a). Barium enema revealed the collapse extending from the descending colon to the rectosigmoid, in addition to the enlarged right-side colon (Fig. 3b). However, the rectum was still able to expand (Fig. 3c). Cine magnetic resonance imaging (MRI) showed weak peristalsis in the enlarged colon, and absent peristalsis in the collapsed colon, but defecography showed normal anorectal function. There was no evidence of systemic diseases causing secondary colonic pseudo-obstruction. Based on the clinical findings and several examinations, we diagnosed this condition as CICP. Since the patient had been hospitalized several times with bowel obstruction and was resistant to medication, we recommended surgical treatment. We suspected that it was caused by defective peristalsis, as the left-side colon was not expanded and had a spastic appearance. Based on the normal anorectal function and the patient's request for curative treatment, we planned to perform laparoscopic total colectomy and ileo-rectal anastomosis (IRA), and avoided permanent ileostomy to ensure postoperative quality of life.

Preoperative fasting and strict defecation control with daily sodium picosulfate hydrate and occasional bisacodyl suppository and glycerin enema were instituted. The day before surgery, colonoscopy was repeated to evacuate the air and stool.

**Fig. 4:**
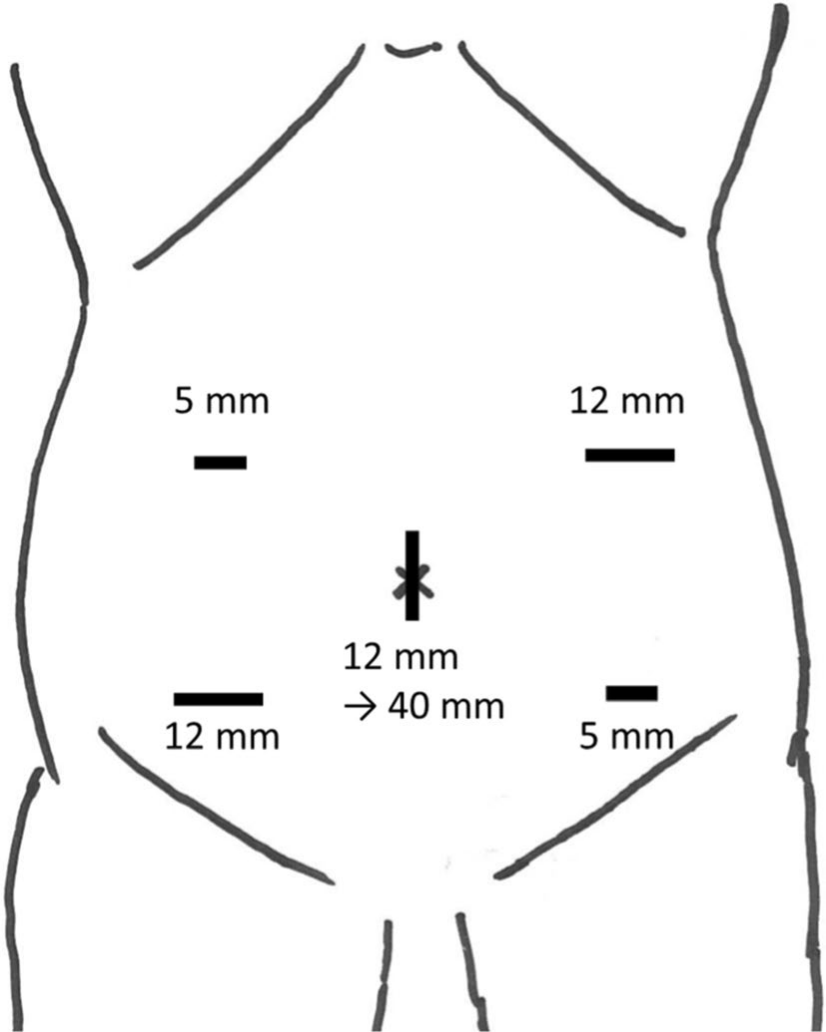
Placement of ports and skin incision for laparoscopic total colectomy. We inserted the laparoscopic trocar and ports as shown in the figure. The umbilical incision was extended to up to 40 mm to remove the colon

Laparoscopic total colectomy was performed under general anesthesia in the lithotomy position. A laparoscopic trocar and ports were inserted as shown in Fig. 4. Mobilization of the right-side colon from the retroperitoneum near the hepatic flexure was started; the ileocecal artery, ileocecal vein, and accessory right colic vein in the mesentery were then divided. The omental bursa was opened, and the splenic flexure mobilized from the retroperitoneum. The right and left branches of the middle colic artery were divided, and the middle colic vein was divided at the main trunk. The inferior mesenteric artery and vein were then divided. The lateral side of the left-side colon was mobilized and attached to the opened omental bursa (proximal to the splenic flexure). The upper rectum was divided using an Endo GIA™ with Tri-Staple™ (Purple 60 mm, Covidien, Minneapolis, MN). The umbilical incision was extended up to 40 mm to remove the colon from the abdominal cavity, and the ileum was divided 10 cm proximal to the ileocecal valve. Thereafter, end-to-end IRA was performed using the double-stapling technique with CDH™ (25 mm, Johnson & Johnson K.K., Ethicon Endo-Surgery Inc., Tokyo, Japan). The operative time was 328 min, and total blood loss volume was 130 mL.

The patient re-started oral feeding on postoperative day 3. She had diarrhea ten times a day, but started experiencing loose stools from around postoperative day 5, without the use of antidiarrheal drugs. She was discharged from the hospital on postoperative day 9 without complications. Five months later, she was defecating solid stools 2–3 times per day, without medication.

**Fig. 5:**
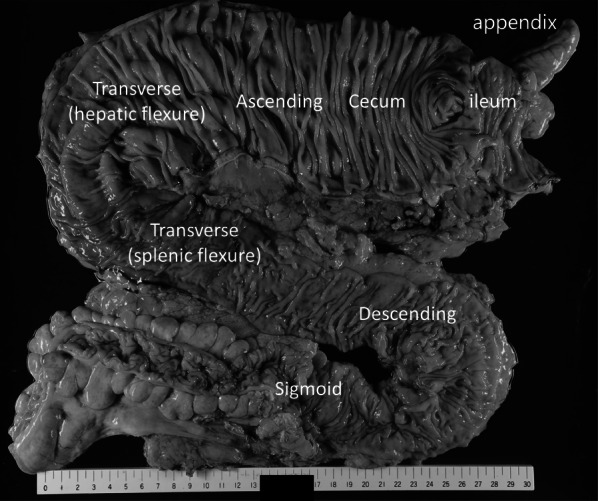
Macroscopic findings. Dilated colon from the cecum to the transverse colon and an abrupt change in diameter in the transverse colon, near the splenic flexure. No abnormal findings detected in the mucosa

**Fig. 6:**
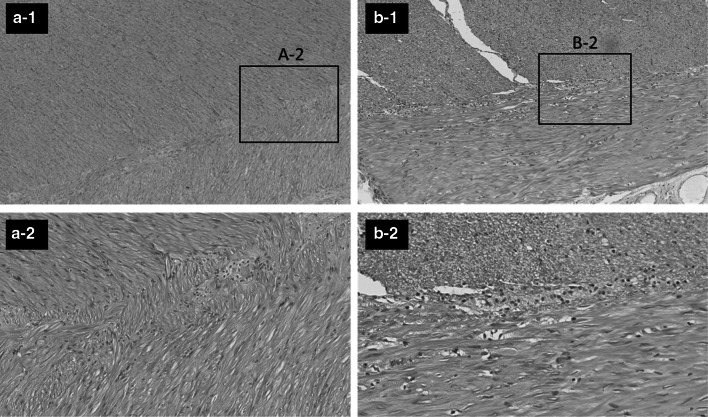
Pathological findings. **a** The muscular layers of the dilated right-side colon (A-1) and its myenteric plexus (Auerbach's plexus) (A-2, HE × 200) show no specific degeneration. **b** Both the longitudinal muscle and the circular muscle in the collapsed left-side colon are thickened, but no vacuolar degeneration is observed (B-1). No specific degeneration is observed in the myenteric plexus (Auerbach's plexus) (B-2, HE × 200)

Dilatation of the colon from the cecum to the transverse colon and an abrupt change in diameter in the transverse colon near the splenic flexure, were observed macroscopically. No abnormal findings were detected in the mucosa (Fig. 5). Pathological examination showed mucosal edema and submucosal fibrosis in the entire length of the resected colon due to chronic inflammation. The dilated right-side colon showed no specific degeneration in the longitudinal muscle, circular muscle, or myenteric plexus (Auerbach's plexus) (Fig. 6a). However, both the longitudinal muscle and the circular muscle in the collapsed left-side colon were thickened. No vacuole degeneration or degeneration in the myenteric plexus (Auerbach's plexus) was observed (Fig. 6b).

## Discussion

In 1958, Dudley et al. reported chronic recurrent abdominal distension, nausea, and vomiting without intestinal stenosis or obstruction as intestinal pseudo-obstruction (IP) [1]. In 1970, Maldonado et al. reported the chronic gastrointestinal disorder of IP with unknown etiology as chronic idiopathic intestinal pseudo-obstruction (CIIP) [2], and in 1986, Anuras et al. reported CIIP with no dysfunction in the small intestine, that is, disease limited to the colon, as chronic idiopathic colonic pseudo-obstruction (CICP) [3]. It is a rare disease in clinical practice, and its etiology, pathophysiology, and prevalence remain poorly understood. The annual rates of CIIP among male and female individuals have been reported to be 0.21/100,000 and 0.24/100,000, respectively [4], but the rates of CICP are unclear. Patients have symptoms, such as bowel obstruction, that persist or recur over 6 months. The differential diagnosis includes Hirschsprung’s disease and acquired isolated hypoganglionosis, among others [5]; however, the diagnosis of CICP must meet the following criteria: (1) dilation of the intestine and abnormal peristalsis limited to the colon, (2) exclusion of mechanical occlusion, (3) exclusion of secondary disorders due to systemic disease (e.g., systemic lupus erythematosus, muscular dystrophy, hypothyroidism, and uremic syndrome, among others) or medication (e.g., antipsychotics, vinca alkaloids, and opioids, among others). In this case, we used multiple imaging modalities to confirm that the defect was limited to the colon. We found the right-side of the colon to be distended, and the left-side to be collapsed with weak peristalsis; however, the small intestine was normal, and the anorectal region demonstrated normal peristalsis. We could not find any occlusion on colonoscopy, and there was no past medical or drug history that could cause organic changes. Based on these clinical and physical findings, we diagnosed the patient to have CICP.

CIIP can cause dysfunction in the gastroduodenum, small intestine, and colon. Unfortunately, there is no effective medication for CIIP. In addition, even if the dysfunctional intestine is resected, intestinal obstruction symptoms may still recur, because dysfunction may exist in the residual intestine. Therefore, conservative treatment with intravenous nutrition is often administered. There is also no effective medication for CICP. It has been reported that evacuating the air or stool by colonoscopy is effective in reducing symptoms [6], but it is only a temporary solution. However, unlike CIIP, there is no dysfunction in the small intestine in CICP. Therefore, surgical resection of the dysfunctional colon is the most common and effective treatment for CICP [7]. Masaki et al. reported that when surgery was performed on 42 patients with CICP, including 23 cases of total colectomy, 90% showed symptomatic improvement [8]. The criteria for selecting either segmental colectomy or total colectomy have not been clarified for CICP. In the case of segmental colectomy, it is possible that symptoms may recur with time, even if they initially improved after surgery. There are some reports of patients that required additional resection years after initial segmental colectomy [9–11]. The reasons included megacolon of unknown cause or ischemic necrosis due to sigmoid colon volvulus. None of them had a definite diagnosis of CICP before the first surgery, but the condition was postoperatively confirmed to be CICP based on the pathological findings. These reports suggest that it is necessary to evaluate the haustra and peristalsis of the remaining colon before additional surgery. It is important to select the appropriate surgical procedure based on several preoperative examinations and the patient’s condition.

In our case, the expandability or peristalsis of the left-side colon was not clear, but functional loss was suspected. We were concerned that similar symptoms may recur if we resected only the expanded right-side of the colon. However, the right-side of the colon demonstrated weak peristalsis; we were also concerned that segmental colectomy of the left-side would not improve the symptoms. To avoid the possibility of multiple surgeries, total colectomy and IRA making use of normal anorectal function were considered appropriate procedures. However, severe diarrhea after surgery was a matter of concern. According to the long-term follow-up of 40 cases of IRA by Fan Li et al., the defecation frequency increased for up to 6 months after surgery, and 8 cases (20%) needed antidiarrheal agents. However, the frequency decreased markedly after that, and only one case (2.5%) required antidiarrheal agents 1 year after surgery [12]. Based on these results, we performed total colectomy and IRA without ileostomy for this patient. Although the follow-up period was only 3.5 years, the patient’s abdominal symptoms have not relapsed, and she is currently not taking any laxative medication.

Unfortunately, we could not identify the cause of the expanded or collapsed right and left sides of the colon based on the pathological findings. We were, therefore, unable to elucidate the etiology of CICP in this case. However, based on the patient’s preoperative condition and postoperative results, our surgical procedure was appropriate. The extent of surgery should not only be selected based on visual findings such as dilatation or collapse, but also on the evaluation of the expandability or peristalsis in both, the colon and rectum.

Laparoscopic surgery is useful in elderly patients, and reduces surgical trauma and postoperative adhesions. It has been reported that laparoscopic colectomy in the elderly is as safe as open surgery, and is a minimally invasive procedure in terms of less bleeding and a shorter length of hospital stay [13]. Certain reports suggest that laparoscopic total colectomy is also a safe procedure in this population [14]. However, in cases of CICP, the dilated colon often causes poor visibility of the surgical field. Decompression of the dilated colon by evacuating gas or stool from the stump of the appendix [10] or transanal drain [15] may improve visibility. In addition, it is possible to secure the operative field using hand-assisted laparoscopic surgery (HALS) if it is difficult to hold down or pull the dilated colon, especially at the splenic flexure [9, 16]. However, reports do not mention the performance of colonoscopy as preparation on the day before surgery, as in our case. Using the preoperative management approach described above, including the evacuation of gas and stool by colonoscopy, we could manipulate the transverse colon in the visible surgical field without using HALS or conversion to laparotomy. The operative time and total blood loss volumes were similar to those reported in previous studies on laparoscopic total colectomy or open/HALS total colectomy [17, 18]. Since the maximum size of the incision was 40 mm, wound pain was well controlled using nonsteroidal anti-inflammatory drugs or acetaminophen. No postoperative complications such as wound infection or paralytic ileus occurred, contributing to the patient’s quick recovery and short hospitalization.

## Conclusions

We present a case of CICP in an elderly patient, who was successfully treated by laparoscopic total colectomy and IRA. We suggest that in addition to the evaluation of the dilated colon, cine MRI and defecography should be used to determine the extent of colectomy. However, continued observation of the patient is required.

## Data Availability

Not applicable.

## Abbreviations

CICP: Chronic idiopathic colonic pseudo-obstruction
IRA: Ileo-rectal anastomosis
HALS: Hand-assisted laparoscopic surgery
CT: Computed tomography
MRI: Magnetic resonance imaging
